# Current Status and Future Prospects of Immune Checkpoint Inhibitors Research in the Treatment of Multiple Myeloma

**DOI:** 10.2174/0118715303379372250417112236

**Published:** 2025-04-29

**Authors:** Xiaojia Guo, Yuan Liu, LianSheng Zhang, Zhengdong Hao, Songlin Chu, Wen Zhou, Lijuan Li

**Affiliations:** 1 Department of Hematology, Lanzhou University Second Hospital, Lanzhou, China;; 2 Department of Pharmacy, Pulmonary Hospital of Lanzhou, Lanzhou, China

**Keywords:** Multiple myeloma, hematological tumor, tumor cells, immune checkpoint inhibitors, immune escape, treatment strategy

## Abstract

Multiple myeloma (MM) is an aggressive hematological tumor characterized by an abnormal proliferation of bone marrow plasma cells accompanied by an overproduction of monoclonal immunoglobulins. The clinical manifestations of MM are varied, with the main ones being anemia, bone pain, renal insufficiency, infections, hemorrhage, neurological symptoms, and hypercalcemia. Chemotherapy, targeted therapy, and stem cell transplantation can provide some relief to patients. However, with the immunodeficiency and immune tolerance that occurs with the progression of the disease, MM is still not completely curable. Immune checkpoint inhibitors (ICIs) provide a new strategy for the treatment of multiple myeloma by blocking the immune escape mechanism of tumor cells and enhancing the ability of T cells to recognize and kill tumors. Currently, immune checkpoint-related therapies such as PD-1/PD-L1, CTLA-4, and TIGIT have attracted extensive attention and research in the field of MM. Due to differences in the genetic characteristics of tumors, the composition of the tumor microenvironment, and the immune status of patients, these therapies face problems such as limited efficacy and immunotoxicity, and the treatment regimens still need to be further optimized. This review summarizes the current research status, challenges and future directions of targeted immune checkpoint therapies for multiple myeloma.

## 1 INTRODUCTION

Multiple myeloma (MM) is a blood cancer that affects blood plasma cells and is characterized by a massive proliferation of tumor plasma cells in the bone marrow, leading to damage to normal blood cells and producing clinical symptoms such as bone pain, anemia and reduced immune system function [1-3]. In recent years, there have been significant advances in treatments for MM, including high-dose chemotherapy, targeted therapies, and stem cell transplants, which are able to offer patients an effective remission. Still, many patients develop drug resistance, leading to eventual relapse [4-6]. Therefore, the search for new therapeutic strategies to reduce drug resistance and improve efficacy is of great importance for patients with multiple myeloma.

Immune checkpoints are signaling molecules that regulate t-cell receptor (TCR) antigen recognition during the immune response and are classified into co-stimulatory and co-suppressive immune checkpoints [7, 8]. They play a regulatory role in the immune system, maintaining immune homeostasis and preventing autoimmune diseases due to over-reactivity [9, 10]. However, tumor cells often use these immune checkpoints to evade the immune system's surveillance and attack [11, 12]. Immune checkpoint inhibitors are a class of drugs that can unlock the suppressive effects of tumor cells on the immune system by targeting specific immune checkpoint molecules, such as PD-1, PD-L1, CTLA-4, *etc*., to enhance T-cell recognition and killing of tumor cells [13-16]. This therapeutic approach has shown significant efficacy in a variety of solid tumors, including melanoma [17, 18], lung cancer [19, 20], and kidney cancer [21, 22]. Several immune checkpoint inhibitors have been used in clinical trials for the treatment of MM, including nivolumab, pembrolizumab, and ipilimumab. They have shown some safety and efficacy in patients with multiple myeloma as monotherapy or in combination with other treatments such as chemotherapy, targeted therapy, or radiotherapy [23-25]. However, immune checkpoint inhibitors have faced some difficulties in the treatment of MM, as due to differences in the genetic characteristics of the tumor, the composition of the tumor microenvironment, and the immune status of the patient, not all patients with MM will respond to these drugs [26]. In addition, immune checkpoint inhibitors may cause some immune-related side effects, such as pneumonia, hepatitis, and colitis [27-30]. To overcome these difficulties, researchers are exploring a variety of strategies, including biomarker discovery and validation to predict patient response to immune checkpoint inhibitors and the development of new combination therapy regimens to improve efficacy and reduce side effects. Immune checkpoint-associated therapies have attracted extensive attention and research in multiple myeloma. This review provides a comprehensive analysis of the clinical use of immune checkpoint inhibitors, and also provides insights into their strengths and limitations in the treatment of multiple myeloma, as well as directions and recommendations for future development in research and clinical practice.

This study is an “Overview” and the manuscript provides a broad summary of the topic area of immune checkpoint inhibitors for multiple myeloma. All literature was accessed through the PubMed database based on the keywords “Multiple myeloma,” “Immune checkpoint inhibitors,” “Immune escape,” and “Treatment strategy” were searched and selected, in addition to clinical data from the National Institutes of Health, URL:clinicaltrials.gov.

## 2 IMMUNOSUPPRESSIVE MICROENVIRONMENT IN MULTIPLE MYELOMA

The multiple myeloma (MM) microenvironment is a complex network of interactions between tumor cells and their surrounding cells and molecules, which plays a key role in tumorigenesis, progression, metastasis, and therapy [31, 32]. In multiple myeloma, tumor cells and myeloid cells inhibit T cell activity by expressing the immune checkpoint molecule PD-L1 to bind to the PD-1 receptor on T cells. The use of PD-1/PD-L1 inhibitors can block this interaction, reactivating T cells and enhancing their ability to recognize and kill tumor cells. In addition to the PD-1/PD-L1 axis, other immune escape mechanisms exist in the multiple myeloma microenvironment, such as an increase in regulatory T cells (Tregs), myeloid-derived suppressor cells (MDSCs), and the secretion of immunosuppressive cytokines [33, 34]. Together, these factors constitute a complex network of immune suppression that makes conventional immunotherapy strategies challenging. The main effect of immune checkpoint inhibitors is to release the inhibitory effect on T cells in the tumor microenvironment and to increase the proliferative capacity, cytokine production, and cytotoxicity of T cells, thereby enhancing the recognition and killing of tumor cells by T cells. In addition, besides directly affecting T cells, immune checkpoint inhibitors are also capable of altering other immune cells in the tumor microenvironment, such as natural killer (NK) cells and dendritic cells [35, 36]. Immune checkpoint inhibitors can block the binding of inhibitory receptors on the surface of NK cells, such as NKG2A, TIM-3, LAG-3, *etc*., to the corresponding ligands on the surface of tumor cells, relieve the immune suppression of NK cells by tumor cells, restore the activity and cytotoxicity of NK cells, and enhance their ability to kill tumor cells. It can also promote the maturation and activation of dendritic cells, and enhance their ability to take up, process and present tumor antigens. T cells can be more effectively activated and initiate specific immune responses.

## 3 CURRENT STATUS OF IMMUNE CHECKPOINT INHIBITORS IN THE TREATMENT OF MULTIPLE MYELOMA

The discovery and study of immune checkpoints have provided new directions and possibilities for immunotherapy of multiple myeloma. Currently, several targets have been applied in immune checkpoint therapy for multiple myeloma, including PD-1, PD-L1, CTLA-4, TIGIT, CD47, LAG-3 and KIR. these targets are key for immune checkpoint inhibitors to work (Fig. **1**). The latest research progress on these immune checkpoints for the treatment of multiple myeloma is summarized below. In addition, we have summarized the current clinical trial data on immune checkpoints for multiple myeloma with reference to CliniclTrial.gov (Table **1**).

### 3.1 PD-1/PD-L1 Inhibitor

PD-1 is a transmembrane protein encoded by the PDCD 1 gene that is expressed on the surface of a wide range of cells [37-39]. PD-L1, a common ligand for PD-1, is present in hematopoietic cells as well as in non-hematopoietic cells, and its expression is regulated by external stimuli [40-42]. T cells in healthy individuals accurately recognize and effectively kill tumor cells. However, in patients with multiple myeloma, PD-1 overexpressed on T cells binds to PD-L1 upregulated on the surface of myeloma cells, causing T cell apoptosis, leading to over proliferation of malignant myeloma cells [43-46]. PD-1/PD-L1 inhibitors can block the PD-1/PD-L1 interaction, reactivate T cells and enhance their ability to recognize and kill tumor cells. Currently, common PD-1 inhibitors include Nivolumab, Pembrolizumab and Pidilizumab, and PD-L1 inhibitors include Avelumab, Atezolizumab and Durvalumab. Clinical studies using Nivolumab for the treatment of patients with relapsed/refractory MM have shown that no significant evidence of the effectiveness of Nivolumab treatment was observed [47]. Pembrolizumab monotherapy has also only supported disease stabilization in patients with relapsed/refractory MM and has not shown significant efficacy. Clinical trials of other monotherapies are currently underway, but available data suggest that monotherapies targeting the PD-1/PD-L1 axis alone have limited efficacy, and that PD-1/PD-L1 inhibitors need to be used in combination with other agents to achieve clinical efficacy. Preclinical studies have shown that lenalidomide increases the effect of PD-1/PD-L1 inhibitors in T lymphocyte and NK cell-mediated cytotoxic effects [48]. A clinical phase I trial of a combination regimen of Pembrolizumab, lenalidomide and low-dose dexamethasone for the treatment of patients with relapsed/refractory MM showed an efficacy rate of 76.5% [49]. However, in patients with multiple myeloma, the combination can also produce some immune toxicity, including hyperthyroidism, colitis and skin reactions [50]. Two phase III randomized trials of pomalidomide and low-dose dexamethasone in combination with or without Pembrolizumab (NCT02576977, NCT02579863) showed that patients with MM may have worsened disease after combined treatment with Pembrolizumab [49]. Therefore, immune toxicity is an important issue that needs to be addressed in the treatment of MM with PD-1 inhibitors in combination with other drugs.

### 3.2 CTLA-4 Inhibitor

CTLA-4 is a T-cell surface transmembrane protein encoded by the CTLA-4 gene, which is homologous to the T-cell co-stimulatory protein CD28 [51-53]. Unlike the CD28 molecule, CTLA-4 is a negative regulator of T-cell activation, inhibiting T-cell activation by binding to the B7 molecule and exerting inhibitory effects by competing for ligands through its extracellular domains to reduce the CD28-mediated stimulatory signals [54]. In addition, CTLA-4 can prevent cell cycle progression by transmitting inhibitory signals through the cytoplasmic tail [55-57]. One study showed that CTLA-4 and FOXP3 were overexpressed in bone marrow samples from patients with MM and suppressed anti-tumor host immune responses [58]. Another study showed that the CTLA-4 genotype identified patients with early progression of MM and was used as a prognostic biomarker [59]. These studies suggest that CTLA-4 plays an important role in the progression of multiple myeloma. CTLA-4 inhibitors enhance the immune system's attack on tumors by blocking the binding of CTLA-4 to CD80/CD86 and lifting the inhibition of T-cell activation. Currently, the common CTLA-4 inhibitors are Ipilimumab and Tremelimumab. Ipilimumab is the first CTLA-4 inhibitor approved by the FDA for the treatment of melanoma and has also been shown to be effective against a variety of hematological malignancies [60]. A clinical study showed that ipilimumab, in combination with nivolumab treatment, had better results in MM patients who relapsed after autologous hematopoietic stem cell transplantation (AHSCT) [25]. A clinical trial of Ipilimumab or nivolumab for use after allogeneic stem cell transplantation was completed, but its results were not available (NCT01822509). There is also currently a clinical trial of ipilimumab in combination with the treatment of myeloma (NCT01592370).

### 3.3 TIGIT Inhibitor

TIGIT, known as T-cell immunoglobulin and ITIM structural domain protein, is an inhibitory receptor mainly expressed in CD8^+^ CTL cells, CD4^+^ Th cells, FOXP3+ Treg cells, and NK cells [61-63]. TIGIT inhibits the immune effects of T cells through a variety of potential mechanisms, and it can directly inhibit T cell activation and proliferation, leading to T cell immune exhaustion, reduced cytokine production, decreased toxicity, and diminished anti-tumor function [64, 65]. High expression of TIGIT on T cells competitively binds the ligands CD155 and CD112 on the surface of myeloma cells or antigen-presenting cells, dephosphorylating CD226 and thus inhibiting anti-tumor effects [66]. In addition, TIGIT can interact with CD155 on dendritic cells, causing immune tolerance in DCs, increasing IL-10 secretion, and decreasing IL-12 secretion, thus exerting an inhibitory effect [62]. TIGIT inhibitors can block the TIGIT signaling pathway, enhance the body's immune response to cancer cells, and increase anti-tumor activity. BMS-986207 is a TIGIT inhibitor. A phase 1/2 trial using the TIGIT inhibitor BMS-986207 for the treatment of relapsed/refractory MM is ongoing (NCT04150965); the clinical efficacy and safety of BMS-986207 alone and in combination with pomalidomide and dexamethasone cannot be evaluated at this time. Autologous Stem Cell Transplantation (ASCT) is an effective therapeutic option for MM patients, and some MM patients achieve long-term disease control with this therapy, but the majority eventually relapse. A preclinical study evaluating the combination of a TIGIT inhibitor and lenalidomide in MM mice after autologous stem cell transplantation (ASCT) showed that the combination of a TIGIT inhibitor and lenalidomide was safe and effective in mice [67]. This suggests that additional synergistic effects may be present in the treatment of MM by TIGIT inhibitors in combination with lenalidomide, which could be subjected to further clinical studies in the future.

### 3.4 CD47 Inhibitor

CD47 is a cell-surface transmembrane protein belonging to the immunoglobulin superfamily that is expressed on the surface of almost all normal cells [68-70]. Integrin, thrombospondin-1, and signal-regulating protein α are the three natural ligands for CD47, which, together with its ligands, are involved in immune-regulatory responses and play an important role in the maintenance of immune homeostasis [71, 72]. In patients with multiple myeloma, CD47 overexpressed on MM cells inhibits phagocytosis by binding to SIRP-a, a signal-regulating protein on the surface of macrophages, releasing a “don’t eat me” signal that allows tumor cells to escape from macrophage immune surveillance [73]. CD47 inhibitors were found to block the binding of CD47 in cancer cells to SIRPα in macrophages, which activates phagocytosis of multiple myeloma cells by macrophages [74, 75]. Currently, common CD47 inhibitors include AO-176, CC-90002, TTI-621 and TTI-622. AO-176 is a humanized IgG2 anti-CD47 mAb that, as a single agent, inhibits tumor growth for a prolonged period in an MM xenograft mouse model [76]. A clinical trial evaluating AO-176 as monotherapy and in combination with bortezomib/dexamethasone in patients with MM was completed, but its clinical results could not be obtained (NCT04445701). CC-90002 is a humanized anti-CD47 antibody that has shown anti-MM tumor activity in preclinical studies [77]. A phase I dose-escalation clinical study has been completed, the results of which are of interest (NCT02367196). TTI-621 and TTI-622 are two soluble recombinant fusion proteins currently in Phase Ia/Ib clinical trials [78, 79]. A clinical trial of TTI-622 administered with bortezomib or carfilzomib + dexamethasone for the treatment of patients with relapsed/refractory MM and lymphoma is ongoing (NCT03530683). In addition, the FDA recently approved a clinical study of the combination of TTI-622, daratumumab, and hyaluronidase-fihj in patients with relapsed/refractory MM (NCT05139225).

### 3.5 LAG-3 Inhibitor

LAG-3 is a type I transmembrane protein with four Ig-like structural domains (D1-D4) that belongs to the immunoglobulin superfamily and is expressed mainly on activated T cells, NK cells, and B cells [80-82]. In immune cells, LAG-3 regulates the activity of T cells, B cells, NK cells, and antigen-presenting cells (APCs), suppresses the immune system response, and maintains the immune homeostasis of the body [83, 84]. A prospective study found that the frequency of PD-1 and LAG-3 positive T cells was strongly associated with the clinical severity of relapsed/refractory MM patients, affecting their prognosis and clinical outcome, and could serve as a potential biomarker for their diagnosis [85]. Another study found that LAG-3 expression was higher on CD4+ Th cells and CD8+ Tc cells in patients with multiple myeloma and that treatment with antibodies targeting LAG-3 significantly enhanced T-cell proliferation and anti-tumor activity [86]. In addition, the expression of GAL-3, a ligand of LAG-3, on MM cells allowed anti-GAL-3 therapy also to increase MM-specific responses [86]. These findings provide a potential therapeutic strategy for LAG-3 blockade, which may be able to enhance antitumor responses and improve the prognosis of patients with MM when used alone or in combination with other agents. Research on LAG-3 inhibitors is still in the early stages. A phase I/II randomized trial of the LAG-3 inhibitor BMS-986016 and the TIGIT inhibitor BMS-986207 as single agents and in combination with pomalidomide and dexamethasone for the treatment of patients with relapsed-refractory multiple myeloma is currently underway (NCT04150965). They are expected to be a new strategy for the future treatment of MM.

### 3.6 KIR Inhibitor

KIR is a family of receptors encoded by polymorphic genes that are mainly expressed in NK cells and some T cells [87, 88]. KIR is divided into KIRxDS-activated and KIRxDL-inhibited types [89]. The inhibitory KIR inhibits the cytotoxic effects of NK when bound to MHC class I ligands on tumor cells, leading to loss of immune surveillance [90, 91]. One study showed that KIR2DL1 expression was higher in myeloma patients compared to healthy controls [92]. Another study showed that progression-free survival in myeloma patients was associated with KIR3DS1 expression [93]. In addition, alterations in the KIR-ligand relationship promote myeloma disease progression [94]. These studies suggest the importance of the KIR with its ligand in myeloma. The main mechanism of action of KIR inhibitors in the treatment of multiple myeloma (MM) is through blocking the interaction of KIR with its ligands, thereby activating NK cells and enhancing their ability to kill tumor cells [89]. Phase I clinical trials of IPH2101, a KIR inhibitor, as monotherapy showed a good safety and tolerability profile, although no significant clinical response rates were observed [95]. Another dose-escalation phase I trial of IPH2101 in combination with lenalidomide in relapsed/refractory myeloma also showed a favorable safety and tolerability profile [96]. Lirilumab (BMS-986015), a second-generation anti-KIR drug from IPH2101, is currently in clinical trials in combination therapy with Elotuzumab and Nivolumab (NCT02252263) (NCT01592370).

## 4 FUTURE CHALLENGES AND PERSPECTIVES

Immune Checkpoint Inhibitors (ICIs) have revolutionized the field of oncology treatment, showing significant efficacy, especially in certain solid tumors and certain hematological cancers. However, the application of ICIs in the treatment of multiple myeloma (MM) faces a number of challenges. Unlike solid tumors, the tumor microenvironment in MM is usually inhibitory to immune cells, and the interaction between tumor cells and immune cells is more complex. MM cells are able to evade surveillance and attack by the immune system through a variety of mechanisms, including down-regulation of the expression of major histocompatibility complex (MHC) molecules and high expression of the immunosuppressive molecule PD-L1 [97, 98]. These factors limit the efficacy of ICIs in MM. The efficacy of ICIs can be enhanced by altering the MM tumor microenvironment by targeting suppressor cells or cytokines. In addition, the development of novel immunomodulatory, such as selective immunomodulatory or small molecule immunomodulatory, could enhance the immune system's attack on MM cells while reducing side effects [99, 100]. Due to factors such as genetic heterogeneity of tumor cells and changes in the immune microenvironment, ICIs treatment may induce an increase in the number or activity of immunosuppressive cells (e.g., Tregs, MDSCs) in the tumour microenvironment. These cells inhibit the immune response through multiple mechanisms, such as Tregs inhibit effector T cell function by paracrine secretion of cytokines such as TGF-β, IL-10, *etc*., and MDSCs deplete arginine by generating arginase, which blocks T cell proliferation and weakens the efficacy of ICIs [101]. The presence of adaptive resistance mechanisms increases the difficulty of treatment and requires the development of new strategies to overcome resistance. To improve the efficacy of ICIs, it is often necessary to combine them with other drugs, such as chemotherapeutic agents, immunomodulatory, and monoclonal antibodies. These combination therapies may also introduce new side effects, and the safety and tolerability of the drugs need to be carefully evaluated. In recent years, immune cell therapies, such as CAR-T cell therapy and NK cell therapy, have shown significant efficacy in certain hematological cancers [102, 103]. ICIs restore and enhance the activity of immune cells, such as T cells and NK cells, by blocking the immune checkpoint signaling pathway. Immune cell therapies enhance the killing ability of immune cells by directly targeting tumor cells. The combination of the two can activate the immune system more comprehensively and improve the killing effect on MM cells. The combination of ICIs and immune cell therapies provides a new strategy and hope for MM treatment. Future studies will further optimize the combination treatment protocol, explore more effective combinations, and verify their safety and efficacy through clinical trials. In addition, there is a lack of validated biomarkers to predict the response of MM patients to ICIs. Tumor mutational burden (TMB) and microsatellite instability (MSI) are indicators of tumor genomic instability, and patients with high TMB and MSI may be more sensitive to treatment with ICIs. In MM, the detection of TMB and MSI can help screen patients suitable for ICI therapy. An in-depth study of the expression patterns, regulatory mechanisms, and relationships with treatment responses of these potential biomarkers will help develop more precise treatment strategies. Meanwhile, combining the detection of multiple biomarkers to establish a comprehensive prediction model will more accurately guide the selection of personalized treatment and combination therapies and improve the therapeutic effect and survival rate of MM patients.

Immune checkpoint inhibitors in the treatment of multiple myeloma, while facing many challenges, also have great potential for development. Through continuous research and innovation, we expect to overcome these challenges and provide more effective and safer treatment options for MM patients. Future research will focus on the discovery of novel immune checkpoints, the development of individualized therapeutic strategies, the modification of the immune microenvironment, the combination of immune cell therapies, the development of novel immunomodulatory, the study of biomarkers, the optimization of clinical trials, the overcoming of drug resistance, and the management of safety and side effects. Through these efforts, we expect to significantly improve the outcome and quality of survival of MM patients.

## CONCLUSION

In recent years, significant progress has been made in immunotherapy for multiple myeloma (MM). The availability of numerous new drugs has significantly prolonged patients' lives; however, MM remains an incurable malignant hematological disease. The development of immune checkpoint inhibitors has provided a new direction in immunotherapy for MM. Such drugs, which fight tumors by enhancing immune surveillance and attenuating inhibitory mechanisms, are essential for MM treatment, especially when combined with novel or conventional therapies, and they may further enhance patients' chances of survival and treatment outcomes. However, drug-induced adverse effects have increased and become a major challenge for their clinical application. We have systematically described the current research situation, challenges and future prospects of immune checkpoint therapy for multiple myeloma, and we hope that more clinical trials can be initiated in the future to validate its efficacy and safety so that immune checkpoint inhibitors can be applied to the clinic as early as possible and the survival prognosis of multiple myeloma patients can be improved.

## Figures and Tables

**Fig. (1):**
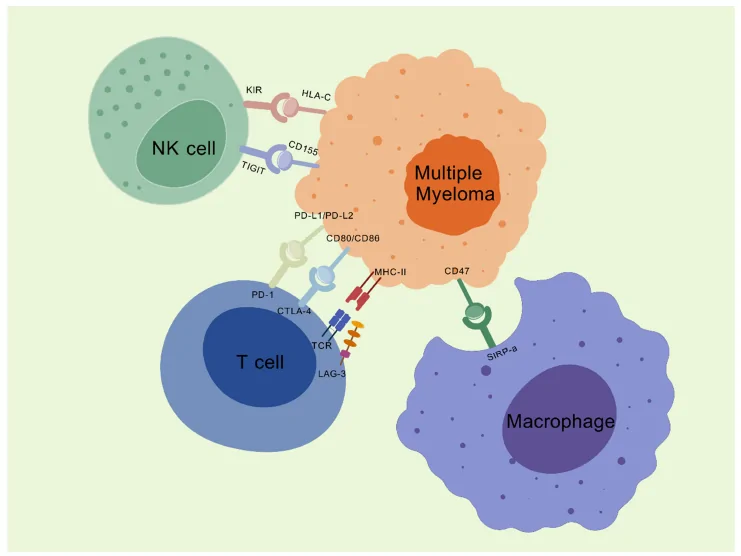
Immune checkpoints in the microenvironment of multiple myeloma. This image was created using the online site https://biogdp.com.

**Table 1:** Current clinical trial data of checkpoint targets and agents.

**Target**	**Agent(s)**	**NCT Number**	**Study Title**	**Phases**	**Enrollment**	**Status**
PD-1	Pembrolizumab	NCT03848845	Study Evaluating Safety, Tolerability and Clinical Activity of GSK2857916 in Combination With Pembrolizumab in Subjects With Relapsed/Refractory Multiple Myeloma (RRMM)	1/2	41	Completed
-	-	NCT02906332	Pembrolizumab + Lenalidomide Post Autologous Stem Cell Transplant (ASCT) in High-risk Multiple Myeloma (MM)	2	12	Terminated
-	-	NCT05191472	Pembrolizumab for the Treatment of Relapsed or Refractory Multiple Myeloma After Anti-BCMA CAR-T Therapies	2	3	Terminated
-	-	NCT05493618	Pembrolizumab, Belantamab and Dexamethasone in Refractory Multiple Myeloma	1/2	0	Withdrawn
-	-	NCT02576977	Study of Pomalidomide and Low Dose Dexamethasone With or Without Pembrolizumab (MK-3475) in Refractory or Relapsed and Refractory Multiple Myeloma (rrMM) (MK-3475-183/KEYNOTE-183)	3	251	Terminated
-	-	NCT03221634	Efficacy and Safety Study of Pembrolizumab (MK-3475) in Combination With Daratumumab in Participants With Relapsed Refractory Multiple Myeloma (MK-3475-668/KEYNOTE-668)	2	0	Withdrawn
-	-	NCT02579863	Study of Lenalidomide and Dexamethasone With or Without Pembrolizumab (MK-3475) in Participants With Newly Diagnosed Treatment Naive Multiple Myeloma (MK-3475-185/KEYNOTE-185)	3	310	Terminated
-	-	NCT03191981	Pembrolizumab Cyclophosphamide and Lenalidomide for Patients With Relapsed Multiple Myeloma	1/2	0	Withdrawn
-	-	NCT02289222	1454GCC: Anti-PD-1 (MK-3475) and IMiD (Pomalidomide) Combination Immunotherapy in Relapsed/Refractory Multiple Myeloma	1/2	48	Terminated
-	Nivolumab	NCT04119336	Nivolumab, Ixazomib, Cyclophosphamide, and Dexamethasone in Relapsed/Refractory Myeloma	2	2	Terminated
PD-1	Nivolumab	NCT03227432	An Exploratory Study to Evaluate the Combination of Elotuzumab and Nivolumab With and Without Pomalidomide in Relapsed Refractory Multiple Myeloma	2	0	Withdrawn
-	-	NCT02903381	A Phase II Trial If Nivolumab, Lenalidomide and Dexamethasone in High Risk Smoldering Myeloma	2	8	Completed
-	-	NCT02612779	A Study of Elotuzumab in Combination With Pomalidomide and Low Dose Dexamethasone and Elotuzumab in Combination With Nivolumab in Patients With Multiple Myeloma Relapsed or Refractory to Prior Treatment With Lenalidomide.	2	74	Completed
-	-	NCT02726581	An Investigational Immuno-therapy Study of Nivolumab, Pomalidomide and Dexamethasone Combinations in Patients With Multiple Myeloma	3	170	Completed
-	Cemiplimab	NCT03194867	Isatuximab in Combination With Cemiplimab in Relapsed/Refractory Multiple Myeloma (RRMM) Patients	1/2	109	Completed
PD-L1	Atezolizumab	NCT03312530	A Study of Cobimetinib Administered as Single Agent and in Combination With Venetoclax, With or Without Atezolizumab, in Participants With Relapsed and Refractory Multiple Myeloma	1/2	49	Completed
-	-	NCT02431208	A Study of Atezolizumab (Anti-Programmed Death-Ligand 1 [PD-L^1^] Antibody) Alone or in Combination With an Immunomodulatory Drug and/or Daratumumab in Participants With Multiple Myeloma (MM)	1	85	Completed
-	Durvalumab	NCT03000452	A Study to Determine the Efficacy of the Combination of Daratumumab (DARA) Plus Durvalumab (DURVA) (D2) in Subjects With Relapsed and Refractory Multiple Myeloma (RRMM)	2	18	Completed
-	-	NCT02685826	A Study of Durvalumab in Combination With Lenalidomide With and Without Dexamethasone in Adults With Newly Diagnosed Multiple Myeloma	1/2	56	Completed
CTLA-4	Tremelimumab	NCT02716805	Phase 1 Study of Tremelimumab, Durvalumab, High-dose Chemotherapy, + Autologous Stem Cell Transplant	1	6	Terminated
-	Ipilimumab	NCT01592370	An Investigational Immuno-Therapy Study to Determine the Safety and Effectiveness of Nivolumab and Daratumumab in Patients With Multiple Myeloma	1/2	316	Completed
-	-	NCT01822509	Ipilimumab or Nivolumab in Treating Patients With Relapsed Hematologic Malignancies After Donor Stem Cell Transplant	1	71	Completed
TIGIT	BMS-986207	NCT04150965	Immuno-Oncology Drugs Elotuzumab, Anti-LAG-3 and Anti-TIGIT	1/2	14	Active, not recruiting
CD47	TTI-622	NCT05139225	A Study of TTI-622 in Combination With Daratumumab Hyaluronidase-fihj in People With Multiple Myeloma	1	7	Active, not recruiting
-	-	NCT03530683	A Clinical Trial to Learn About the Study Medicine Called Maplirpacept (PF-07901801), Alone and When Used in Combination With Other Medicines to Treat Participants With Advanced Hematological Malignancies, Including Lymphoma, Leukemia and Multiple Myeloma	1	189	Terminated
-	AO-176	NCT04445701	Study of AO-176 as Monotherapy and in Combination With Bortezomib/Dexamethasone in Relapsed/Refractory Multiple Myeloma	1/2	10	Completed
-	CC-90002	NCT02367196	A Phase 1, Dose Finding Study of CC-90002 in Subjects With Advanced Solid and Hematologic Cancers	1	60	Completed
LAG-3	BMS-986016	NCT04150965	Immuno-Oncology Drugs Elotuzumab, Anti-LAG-3 and Anti-TIGIT	1/2	14	Active, not recruiting
KIRs	IPH2101	NCT00999830	Evaluation of Activity, Safety and Pharmacology of IPH2101 a Human Monoclonal Antibody in Patients With Multiple Myeloma	2	27	Completed
-	-	NCT01222286	Study on the Anti-tumor Activity, Safety and Pharmacology of IPH2101 in Patients With Smoldering Multiple Myeloma	2	30	Completed
-	-	NCT01217203	Study on the Safety, Anti-tumor Activity and Pharmacology of IPH2101 Combined With Lenalidomide in Patients With Multiple Myeloma Experiencing a First or Second Relapse	1	15	Completed
-	-	NCT00552396	An Open-label, Dose-escalation Safety and Tolerability Trial Assessing Anti-KIR (1-7F9) in Subjects With Multiple Myeloma	1	32	Completed
-	-	NCT01248455	A Phase II Trial of Anti-KIR in Smoldering Multiple Myeloma	2	9	Terminated
-	Lirilumab	NCT02252263	A Phase I Open Label Study of the Safety and Tolerability of Elotuzumab (BMS-901608) Administered in Combination With Either Lirilumab (BMS-986015) or Urelumab (BMS-663513) in Subjects With Multiple Myeloma	1	44	Completed
-	-	NCT01592370	An Investigational Immuno-Therapy Study to Determine the Safety and Effectiveness of Nivolumab and Daratumumab in Patients With Multiple Myeloma	1/2	316	Completed

## LIST OF ABBREVIATIONS

ICIs: Multiple Myeloma
ICIs: Immune Checkpoint Inhibitors
MDSCs: T-cell Receptor
MDSCs: Myeloid-Derived Suppressor Cells
NK: Natural Killer
RRMM: Refractory Multiple Myeloma
AHSCT: Autologous Hematopoietic Stem Cell Transplantation
ASCT: Autologous Stem Cell Transplantation
APCs: Antigen-presenting Cells
TMB: Tumor Mutational Burden
MSI: Microsatellite Instability
